# Untargeted Metabolomics via UPLC-HRMS and ASCA-Based Statistical Modeling to Characterize Metabolic Alterations Following Acute and Repeated Brorphine Exposure

**DOI:** 10.3390/metabo16100770

**Published:** 2026-10-12

**Authors:** Ilenia Bracaglia, Ludovica Chiodo, Camilla Montesano, Sabrine Bilel, Marta Bassi, Nicole Cocita, Fabiana Di Rosa, Adolfo Gregori, Matteo Marti, Federico Marini, Manuel Sergi

**Affiliations:** 1 Department of Chemistry, Sapienza University of Rome, 00185 Rome, Italy; ilenia.bracaglia@uniroma1.it (I.B.); ludovica.chiodo@uniroma1.it (L.C.); federico.marini@uniroma1.it (F.M.); manuel.sergi@uniroma1.it (M.S.); 2 Department of Public Health and Infectious Disease, Sapienza University of Rome, 00185 Rome, Italy; 3 Department of Environmental Sciences and Prevention, University of Ferrara, 44121 Ferrara, Italy; bllsrn@unife.it; 4 Department of Translational Medicine, Section of Legal Medicine and LTTA Centre, University of Ferrara, 44121 Ferrara, Italy; marta.bassi@unife.it (M.B.); nicole.cocita@unife.it (N.C.); mto@unife.it (M.M.); 5 Carabinieri, Department of Scientific Investigation (RIS), 00191 Rome, Italy; fabiana.dirosa@carabinieri.it (F.D.R.); adolfo.gregori@carabinieri.it (A.G.); 6 Collaborative Center for the Italian National Early Warning System (NEWS-D), Department of Anti-Drug Policies and Other Addictions, Presidency of the Council of Ministers, 00184 Rome, Italy

**Keywords:** novel synthetic opioids, brorphine, untargeted metabolomics, UPLC-HRMS, ASCA, sex differences, forensic toxicology, new psychoactive substances

## Abstract

**Background:** Novel Synthetic Opioids (NSOs) currently represent the third most prevalent group among New Psychoactive Substances (NPS), yet they represent the category associated with the highest health risk. Their extreme pharmacological potency compared to traditional opioids, coupled with structural heterogeneity and low concentrations in biological matrices, poses significant analytical challenges for detection. **Methods:** This study proposes an indirect screening approach based on untargeted metabolomics to monitor systemic responses following brorphine administration. Urinary metabolic profiling was conducted using a sex-balanced murine cohort (*n* = 10); metabolic profile variations were analyzed following acute and repeated administrations. Samples were collected at 0–12 h and 12–24 h intervals and analyzed by liquid chromatography coupled with high resolution mass spectrometry (LC-HRMS); data were processed using ANOVA–Simultaneous Component Analysis (ASCA), for the isolation of metabolic fingerprints associated with distinct experimental factors (sex, treatment, sampling time). **Results:** The results revealed a notable metabolic divergence upon repeated administration, linked to sexual diergism: while females exhibited a high recovery capacity supported by a more efficient metabolic biotransformation, males showed a stronger energetic deficit, likely due to greater substance persistence and accumulation. Male vulnerability is characterized by neuro-metabolic dysfunction, inhibition of γ-aminobutyric acid (GABA) release and altered dopaminergic turnover. This systemic imbalance is further defined by massive amino acid and nucleotide catabolism, alongside an energetic deficit exacerbated by inefficient *β*-oxidation. **Conclusions:** This study demonstrates that metabolomics may provide interesting insights for the study of NPS by recognizing the biochemical signatures of drug consumption and it is essential for developing sex-specific strategies within the broader context of opioid pharmacology.

## 1. Introduction

In forensic toxicology, New Psychoactive Substances (NPS) have emerged as a primary global public health concern. Their high toxicological potency, structural heterogeneity [1,2], low concentrations in biological matrices and often short biological half-lives complicate their analytical detection and identification [3], thereby hindering effective regulatory control.

Data reported in the World Drug Report 2024 [4] and confirmed in 2025 [5] indicate that, although the overall number of identified NPS shows a decreasing trend, there is a significant increase in Novel Synthetic Opioids (NSO). NSOs currently represent the third largest group among NPS, following synthetic cathinones and synthetic cannabinoids and are considered the class associated with the highest health risk, owing to their extremely high pharmacological potency compared to traditional opioids [6,7]. Such high potency is frequently associated with severe depressive effects on the central nervous system (CNS) and respiratory function [8], which may also persist over the long term, in relation to their metabolic biotransformation into pharmacologically active metabolites [9], as well as with a high risk of accidental overdose, even at low doses [10]. Beyond their acute toxic effects, chronic opioid exposure leads to neuroadaptive changes that underlie tolerance and dependence, characterized by alterations in intracellular signaling pathways, receptor sensitivity, and synaptic transmission [11,12].

Chemically, NSOs are categorized into fentanyl analogues and non-fentanyl synthetic opioids [13]. The latter group includes heteropolycyclic amine derivatives, particularly benzimidazoles, among which nitazenes represent the most relevant emerging group, known for a potency up to 1000 times that of morphine [14], cyclohexylbenzamides and benzimidazolones, a class that includes brorphine and its derivatives [15]. Although brorphine was first described in the literature in the 1960s, its market prevalence increased in tandem with the decline in nitazene consumption in the United States during 2020 [16,17]. Several studies have further highlighted significant variations in μ-opioid receptor (MOR) agonist selectivity among brorphine analogues [18]. The clinical danger of brorphine is critically compounded by frequent polydrug use; in fact, it is often detected in combination with other opioids or benzodiazepines, particularly flualprazolam [16,17], which further increases overall toxicity.

In this context, metabolomics has become of fundamental importance, playing a crucial role in recent years also within the field of precision medicine, as it enables the identification of potential diagnostic, prognostic and therapeutic biomarkers [19,20]. It allows the detection of metabolic alterations and characteristic profiles associated with different phenotypes, such as consumers of NPS, allowing for the assessment of acute and chronic effects, including mechanisms of dependence, tolerance, and withdrawal, despite the limited availability of clinical data [21,22,23,24].

Given the intrinsic complexity of this field, advanced analytical workflows combining untargeted metabolomics with multivariate statistical analysis are required [25]. These approaches are further enhanced by in silico prediction tools and retrospective data mining to interpret the complex dataset generated by LC-HRMS analyses, one of the most widely used analytical platforms in this context due to its high sensitivity, broad coverage of small molecules and its capability to confirm the identity of metabolites of interest [26]. Ultimately, a rigorous experimental design is the essential foundation for ensuring the validity and reproducibility of metabolomic studies [27].

In this study, an in vivo murine model was employed, in which male and female mice received acute and repeated administrations of brorphine. Urine samples, collected at two different time intervals, before and after drug administrations, were analyzed by LC-HRMS using an untargeted approach. This strategy aimed to evaluate variations in endogenous urinary metabolite profiles, reflecting the systemic responses to substance exposure. Subsequently, the resulting data were subjected to multivariate statistical analysis to identify biochemical signatures associated with brorphine intake. First, Principal Component Analysis (PCA) was performed for exploratory data analysis and dimensionality reduction, focusing on the treatment factor (vehicle-treated vs acutely drug-treated samples). Next, Partial Least Squares Discriminant Analysis (PLS-DA) was applied to enhance group discrimination and identify the most relevant variables based on Variable Importance in Projection (VIP) scores. Finally, ANOVA–Simultaneous Component Analysis (ASCA) was performed on the entire dataset, including all experimental conditions, to investigate characteristic metabolic fingerprints associated with each experimental factor (sex, acute and repeated treatment, sampling time), while minimizing the number of false positives commonly observed in large-scale datasets [27]. ASCA has been successfully applied in diverse -omics fields for its ability to interpret complex, non-linear longitudinal trends and to elucidate the contribution of experimental design factors and their interactions to the overall variability of the dataset [28,29].

## 2. Materials and Methods

### 2.1. Chemicals and Reagents

Brorphine was purchased from Cayman Chemical (Ann Arbor, MI, USA). The drug was initially dissolved in absolute ethanol (final concentration: 5%) and Tween 80 (2%) and brought to the final volume with saline (0.9% NaCl). Brorphine was administered by intraperitoneal (IP) injection at a volume of 4 μL/g. The final concentration of brorphine was 15 mg/kg. Ultrapure water LC-MS grade was obtained by a Milli-Q Plus Millipore apparatus (Bedford, MA, USA), ammonium formate, acetonitrile (AcN), methanol (MeOH) and formic acid (HCOOH) were acquired from Fluka Bio Chemika Sigma-Aldrich (Milan, Italy). Morphine, Cocaine, Δ9-tetrahydrocannabinol (THC), Δ9-tetrahydrocannabinolic acid A (THC-A), cannabidiolic acid (CBD-A) and ethyl glucuronide (ETG) were purchased from Sigma–Aldrich (Burlington, MA, USA) and used as internal standards (IS); the working IS solutions were prepared at a final concentration of 300 ng/mL by dilution using different solvent systems according to the chromatographic mode, namely AcN for reversed-phase (RP) analysis and ultrapure water for hydrophilic interaction liquid chromatography (HILIC) analysis.

All analytical solutions were stored at −20 °C.

### 2.2. In Vivo Study on Mice

The study was conducted using outbred ICR (CD-1) mice, a strain widely used in pharmacological and toxicological research and previously employed in our investigations of NPS. Its use in the present study was consistent with our established experimental model and facilitated comparison with our previous investigations of synthetic opioids. Both male and female mice were included to enable the assessment of potential sex-related differences in brorphine metabolism and urinary pharmacokinetics. Specifically, ten young adult ICR (CD-1) mice (8–10 weeks old; 5 males and 5 females) were individually housed in metabolic cages (Ugo Basile SRL, Gemonio [VA], Italy) with free access to water and food in a colony room under constant temperature (23–24 °C) and humidity (45–55%). The group of ten animals, equally balanced by sex, was subjected to different treatments according to the experimental protocol [30,31]. The experimental protocols performed in the present study were in accordance with the U.K. Animals (Scientific Procedures) Act of 1986 and associated guidelines and the new European Communities Council Directive of September 2010 (2010/63/EU). Experimental protocols were approved by the Italian Ministry of Health (license No. 223/2021-PR, CBCC2.46.EXT.21, approved on 22 March 2021) and by the Animal Welfare Body of the University of Ferrara. According to the ARRIVE guidelines, all possible efforts were made to minimize the number of animals used, to minimize the animals’ pain and discomfort and to reduce the number of experimental subjects.

**Figure 1:**
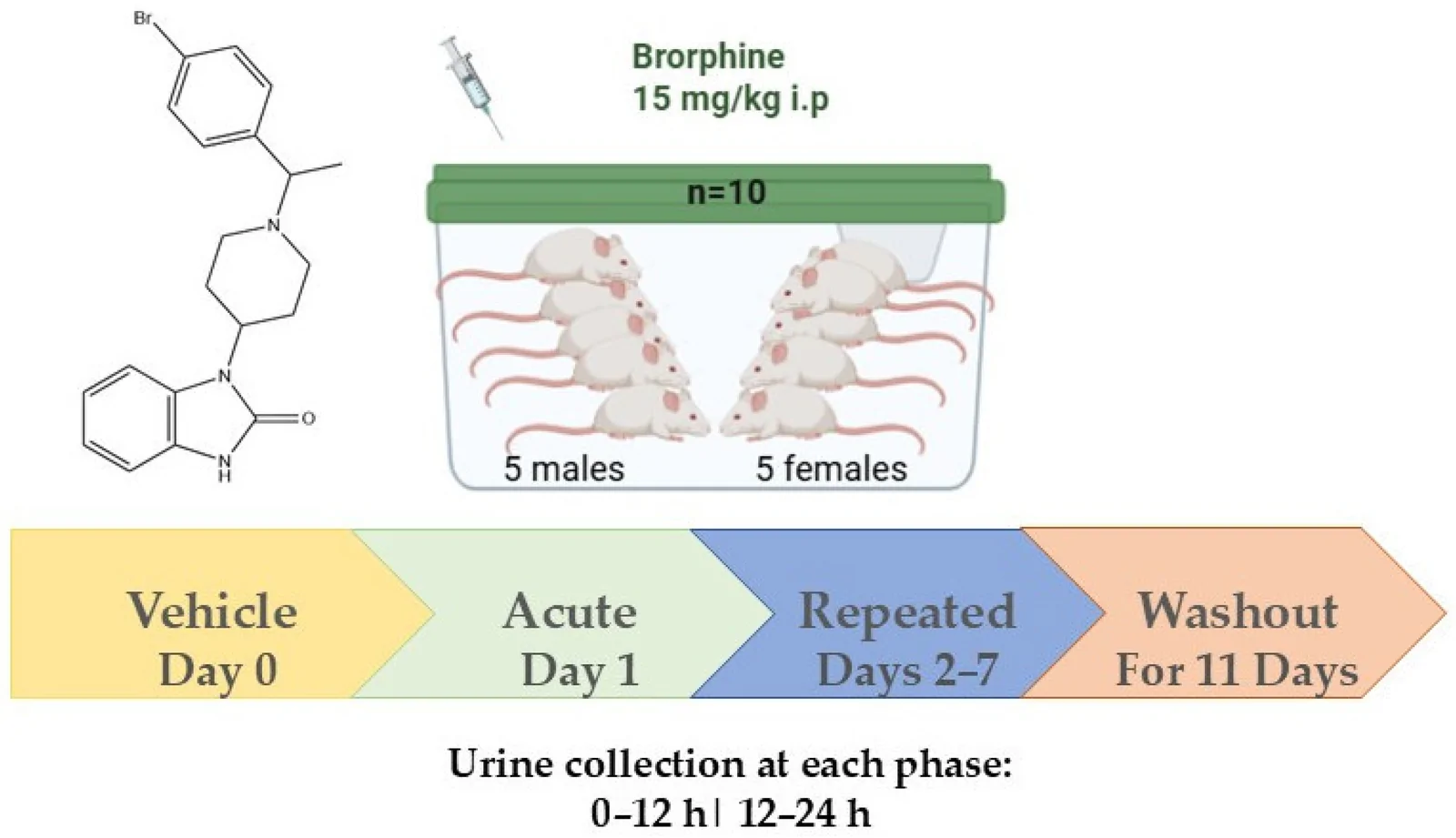
Experimental timeline of the in vivo metabolomic study and brorphine chemical structure.

The metabolomic study design (Figure 1) consisted of the following steps. The same animals were followed longitudinally throughout all experimental phases. On day 0 (vehicle/baseline phase), mice received an intraperitoneal injection of vehicle, and urine was collected over two consecutive intervals (0–12 h and 12–24 h). Then, all animals received daily intraperitoneal injections of brorphine (15 mg/kg) for seven consecutive days. Using the same two collection intervals, urine was then collected from the same animals at three further time points: after acute exposure (first administration, day 1), after repeated exposure (last administration, day 7), and after an 11-day washout period. The samples were then stored at −20 °C until analysis.

### 2.3. Sample Preparation

#### 2.3.1. Urine Sample Preparation

Urine samples were diluted 1:10 (*v*/*v*) with ultrapure water or acetonitrile for RP and HILIC analyses, respectively, and spiked with internal standards.

#### 2.3.2. Quality Control Samples and Blanks

Two types of quality control (QC) samples were prepared, namely, pooled QCs and diluted QCs, which were used for metabolite signal normalization and assessment of MS signal linearity, respectively [32]. The pooled QC was prepared by combining 5 µL of each available urine sample. The resulting pooled QC was then pre-treated identically as the study samples, undergoing a 1:10 (*v*/*v*) dilution with ultrapure water or acetonitrile for RP and HILIC analyses, respectively, followed by further dilution after the addition of the internal standard mixture. Pooled QC samples were injected (*n* = 10) at the beginning of the sequence to condition the LC–MS system and subsequently injected every eight real samples throughout the batch for system monitoring and signal normalization. Diluted QCs were prepared by diluting 2, 4, and 8 folds the pooled QC with ultrapure water or acetonitrile, according to the chromatographic mode, and were analysed three times per analytical run to evaluate the linearity of the MS response.

Blank samples consisted of ultrapure water for RP analysis and acetonitrile for HILIC analysis and were injected three times at the beginning of the analytical sequence to acquire background signals, which were subsequently excluded from the dataset.

### 2.4. UPLC-HRMS Analysis

#### 2.4.1. Chromatographic Separation

An Ultimate 3000 RSLC system coupled with a Q-Exactive Mass spectrometer (Thermo Fisher Scientific, Bremen, Germany) was used for analysis.

Reverse Phase (RP) chromatographic separation was performed on a Kinetex F5 column (100 × 2.1 mm i.d., 2.6 µm particle size; Phenomenex), the column oven was maintained at 35 °C. The mobile phase flow rate was set to 0.300 mL min^−1^. The mobile phases consisted of water containing 0.1% (*v*/*v*) formic acid (Phase A) and acetonitrile containing 0.1% (*v*/*v*) formic acid (Phase B). The gradient started at 0% B, held for 1.5 min, then increased linearly to 10% B at 4.0 min, 35% B at 7.0 min and 98% B at 10.0 min. These conditions were maintained until 12.0 min, after which the system was returned to the initial conditions (0% B) and re-equilibrated until 15.0 min, resulting in a total run time of 15 min.

Hydrophilic Interaction Chromatography (HILIC) separation was performed on a Kinetex HILIC column (100 × 2.1 mm i.d., 2.6 µm particle size; Phenomenex) the column oven was maintained at 35 °C. The mobile phase flow rate was 0.500 mL min^−1^. The mobile phases consisted of water containing 0.1% (*v*/*v*) formic acid and 20 mM ammonium formate (Phase A) and acetonitrile containing 0.1% (*v*/*v*) formic acid (Phase B). The gradient started at 95% B, held for 3.5 min, then decreased linearly to 75% B at 8.5 min, 40% B at 9.5 min, and 0% B at 11.5 min. Phase B was then rapidly increased back to 95% at 15.0 min to allow column re-equilibration, resulting in a total run time of 15 min.

For both analyses, the injection volume was 2 µL.

#### 2.4.2. High-Resolution Mass Spectrometry Analysis

The Q-Exactive mass spectrometer was equipped with a heated electrospray ionization source (HESI-II) operated in both positive and negative modes; mass spectra were acquired in full scan for all samples while pooled QC were also analysed in full scan/data dependent (DDA) in the range 50–650 *m*/*z*. For RP analysis, the spray voltage was set to 3.20 kV in positive-ion mode and −3.20 kV in negative-ion mode. The capillary temperature was 350 °C, the HESI heater temperature was 300 °C, and the S-lens RF level was set to 50 (positive mode) and −50 (negative mode). The sheath gas flow rate was 40, while the auxiliary gas flow rate was 15. For HILIC analysis, the spray voltage was set to 2.50 kV in positive-ion mode and −2.50 kV in negative-ion mode. The capillary temperature was 288 °C, the HESI heater temperature was 300 °C, and the S-lens RF level was set to 50 (positive mode) and −50 (negative mode). The sheath gas and auxiliary gas flow rates were set to 60 and 18, respectively. Nitrogen was used as spray stabilization gas, as the collision gas for collision-induced dissociation experiments in the high-energy collision dissociation (HCD) cell and as damping gas in the C-trap.

In full-scan mode, the resolution was 70,000 (FWHM at *m*/*z* 200), whereas the automatic gain control (AGC) and maximum injection time were set to 1 × 10^5^ and 100 ms, respectively. In MS/MS mode, resolution was 17,500 (FWHM at *m*/*z* 200) and three different collision energies, i.e., 10, 30, 50 eV, were applied. For DDA, three different runs were acquired with different acquisition windows: 50–200 *m*/*z*, 200–400 *m*/*z* and 400–650 *m*/*z*, respectively.

The instrument was calibrated daily in both positive and negative ion modes.

### 2.5. Statistical Analysis

#### 2.5.1. Data Preprocessing

The UPLC-HRMS raw files acquired in positive and negative ionization modes were processed separately using Compound Discoverer 3.2 (Thermo Scientific, Waltham, MA, USA). The data processing followed the non-targeted metabolomics workflow previously optimized by our research group [32], regarding retention time alignment, component detection, elemental composition prediction, and gap filling. Briefly, peak picking, unknown compound detection, blank subtraction, QC-based filtering and generation of the output feature table were performed according to these standardized parameters. This procedure generated an output table containing *m*/*z*, retention time, and peak intensity values, together with QC-normalized peak areas for each detected metabolic feature. Specifically, the process resulted in four datasets, the first two, acquired in RP, contained 154,000 features; the last ones, acquired using the HILIC, with 85,000 features.

Datasets generated by Compound Discoverer were exported as .xls files and imported into MATLAB version R2024a (The MathWorks, Natick, MA, USA). As previously described [32], two sequential filtering strategies were applied to reduce the number of detected features subjected to subsequent statistical analysis: (i) a QC-based dilution series filtering to retain reliable features [33,34] and (ii) a Pearson correlation-based clustering approach to group ions originating from the same parent compound (adducts, dimers, fragments, isotopes), retaining only the most abundant feature within each cluster [35]. Finally, all four data sets (RP HESI+, RP HESI−, HILIC HESI+, HILIC HESI−) were normalized using probabilistic quotient normalization (PQN) [36,37] in order to correct differences in urine dilution.

#### 2.5.2. Principal Component Analysis (PCA) and Partial Least Squares Discriminant Analysis (PLS-DA)

PLS Toolbox version 9.3.1 (Eigenvector Technologies, Manson, MA, USA) was used to perform PCA, while PLS-DA and ASCA were carried out by means of in-house written functions running under the MATLAB environment. Prior to analysis, data were first subjected to autoscaling, and successively, block-scaling was applied to the individual data matrices prior to merging the four datasets according to a low-level fusion protocol, yielding a total of 31,217 features. Brorphine and putatively identified metabolite features were removed from the dataset to obtain a more reliable multivariate statistical analysis of the endogenous data.

PCA was employed for preliminary data exploration to provide an initial assessment of treatment-related variability, i.e., to assess whether vehicle-treated and acutely drug-treated samples exhibit a certain extent of separation, already at an unsupervised level. Subsequently, PLS-DA was employed as a supervised classification method to model the relationship between the measured variables and treatment class, enabling discrimination between vehicle-treated and acutely drug-treated samples and the identification of the most influential variables through VIP scores. Following multivariate analysis, a delta matrix (Δ) was constructed to isolate treatment-induced metabolic responses while minimizing inter-individual baseline variability. To this end, all experimental conditions were taken into account, including acute drug administration, repeated administration (7 days), and the washout phase. Specifically, for each animal, the normalized peak area of each feature measured in the corresponding vehicle sample was subtracted from that obtained after drug administration, for each treatment condition and sampling interval (0–12 h and 12–24 h), so that each animal served as its own control. This subject-wise differencing is conceptually analogous to the within-subject decomposition adopted in multilevel analysis, as it removes baseline inter-individual differences and retains only the metabolic changes attributable to treatment. The resulting Δ matrix was subsequently analysed using ASCA to partition the structured variability associated with the experimental factors of interest, namely sex, sampling interval, and treatment stage (acute administration, repeated administration, and washout), as well as their interactions.

#### 2.5.3. ANOVA–Simultaneous Component Analysis (ASCA)

ASCA enables the identification of the variables responsible for the differences observed among the levels, the experimental factors and their interactions by combining the ANOVA decomposition with multivariate analysis. Specifically, the variance of the data matrix is partitioned, according to the ANOVA framework, into contributions associated with the main effects and interactions defined by the experimental design, together with a residual matrix accounting for the unexplained variability [29,38]. Each contribution is represented by an effect matrix, containing the mean structure associated with the corresponding factor or interaction.

The interpretation of these contributions is achieved by applying Simultaneous Component Analysis (SCA), which, under the ANOVA constraints, reduces to an ordinary PCA of the corresponding effect matrix [39]. Unlike ANOVA-PCA, where the residual matrix is added to each effect matrix before PCA, ASCA applies SCA directly to the individual effect matrices, thereby avoiding the confounding of different sources of variation and facilitating the biological interpretation of the experimental effects [40]. The resulting score vectors describe the relationships among the factor levels, whereas the loading vectors identify the variables contributing most strongly to each effect [41].

In the present study, ASCA was applied to the difference matrix (ΔX) described above, in which, for each animal, treatment condition and sampling interval, the corresponding vehicle profile was subtracted from the measured metabolic profile. Thus, the analysed responses represented within-animal metabolic changes relative to vehicle rather than the original absolute metabolomic profiles. Prior to ASCA, variables of the ΔX matrix were autoscaled to unit variance to prevent differences in feature scale from dominating the multivariate decomposition.

The experimental design included one between-subject factor, sex (male and female), and two within-subject factors, treatment stage (acute administration, repeated administration and washout) and sampling interval (0–12 h and 12–24 h). Because multiple observations were obtained from the same animals and not all combinations of the within-subject factors were available for every subject, the resulting design was both repeated-measures and unbalanced. Consequently, the ASCA decomposition was implemented within a general linear model (GLM) framework rather than by deriving effect matrices directly from marginal means.

Let ΔX denote the (N × J) matrix of within-animal metabolic differences, where N is the number of available observations and (J) is the number of metabolic features. The fixed-effects model included the three main experimental factors and all their two- and three-way interactions:

(1) ΔX = 1*m^T^* + *X_sex_* + *X_treatment_* + *X_samplingtime_* + *X_sex_*_×_*_treatment_* + *X_treatment_*_×_*_samplingtime_* + *X_sex_*_×_*_sampling_*_time_
+ *X_sex_*_×_*_treatment_*_×_*_sampling_*_time_ + *X_residuals_*

where *sex*, *treatment*, and *samplingtime* denote sex, treatment stage, and sampling interval, respectively; *m* represents the overall mean profile; the *X_k_* terms represent the multivariate contributions associated with the corresponding main effects or interactions; and *X_residuals_* is the residual matrix. Subject identity was not included as an additional fixed main effect because the response matrix had already been expressed as within-animal differences relative to the corresponding vehicle measurements. Subject identity was, however, retained to define the repeated-measures structure and the restrictions applied during permutation testing.

Because the experimental design was unbalanced, the contributions associated with the different model terms are not necessarily mutually orthogonal, and their sums of squares are therefore not assumed to provide an additive partition of the total sum of squares. Each experimental term was instead evaluated using Type III sums of squares, i.e., its contribution was estimated after adjustment for all the other terms included in the model. Let *H_F_* denote the projection matrix associated with the full GLM and *H_R_*_,_*_k_* the projection matrix of the corresponding reduced model obtained by excluding term (k) while retaining all remaining model terms. The Type III adjusted effect matrix for term (k) was calculated as

(2) $$X_{k}^{\mathrm{III}}={(H}_{F}-H_{R,k})\Delta X$$

and the magnitude of the corresponding effect was quantified by its Type III sum of squares:

(3) $${\mathrm{SSQ}}_{k}={\left\|X_{k}^{\mathrm{III}}\right\|}_{F}^{2}$$

where ${\left\|\cdot \right\|}_{F}^{2}$ denotes the Frobenius norm. This formulation allows the unique contribution of each main effect or interaction to be evaluated conditionally on the remaining terms of the model and is therefore suitable for the unbalanced experimental design.

Statistical significance was assessed using factor-specific restricted permutation schemes designed to preserve the repeated-measures structure of the experiment. For within-subject effects, a Freedman–Lane residual permutation procedure was adopted. Residuals were permuted exclusively among observations belonging to the same animal, so that observations were never exchanged between different biological subjects. This procedure preserves the fitted contributions of the nuisance terms contained in the reduced model while randomizing the residual component relevant to testing the effect under investigation.

For effects involving sex, which represents a between-subject factor, randomization was instead performed at the subject level. Sex labels were permuted among complete biological subjects while all repeated observations belonging to the same animal were kept together. Thus, the repeated-measures structure was preserved, and no individual observations were exchanged independently between animals.

Repetition of this procedure generated an empirical null distribution of the Type III sum of squares for each experimental effect. This restricted randomization strategy therefore allowed the statistical significance of the experimental effects to be assessed while accounting simultaneously for the unbalanced design and the dependence among repeated observations from the same animal.

Each statistically significant Type III effect matrix was subsequently analysed by Simultaneous Component Analysis (SCA) to characterize its multivariate structure.

SCA corresponds to a principal component decomposition of the adjusted effect matrix ( $X_{k}^{\mathrm{III}}$ ) was decomposed into scores (*T_k_*) and loadings (*P_k_*) matrices:

(4) $$X_{k}^{\mathrm{III}}=T_{k}P_{k}^{T}+E_{k}$$

Variables contributing significantly to each experimental effect were identified using a bootstrap procedure applied to the corresponding loading vectors [41]. Confidence intervals were estimated for each loading coefficient, and variables whose confidence interval did not include zero were considered relevant contributors to the corresponding multivariate effect. Importantly, the permutation tests described above were performed at the level of the overall multivariate experimental effects and interactions, using the Type III sum of squares of each effect matrix as the test statistic, rather than separately for each metabolic feature. Consequently, the resulting permutation *p*-values do not constitute feature-wise multiple hypothesis tests, and FDR correction was not applied to the ASCA permutation *p*-values. Identification of the individual variables contributing to significant effects was instead based on the bootstrap confidence intervals of the corresponding loading coefficients.

## 3. Results

### 3.1. Multivariate Analysis—Vehicle vs. Single Administration

The integration of the four analytical platforms (RP HESI+, RP HESI−, HILIC HESI+, HILIC HESI−) provided extensive coverage of the urinary metabolome, encompassing both non-polar and polar species. As detailed in the materials and methods section, the application of rigorous filtering, normalization (PQN), and data pretreatment (scaling) stages was fundamental to balance the contribution of variables across different intensity scales and to minimize the impact of analytical noise [42].

**Figure 2:**
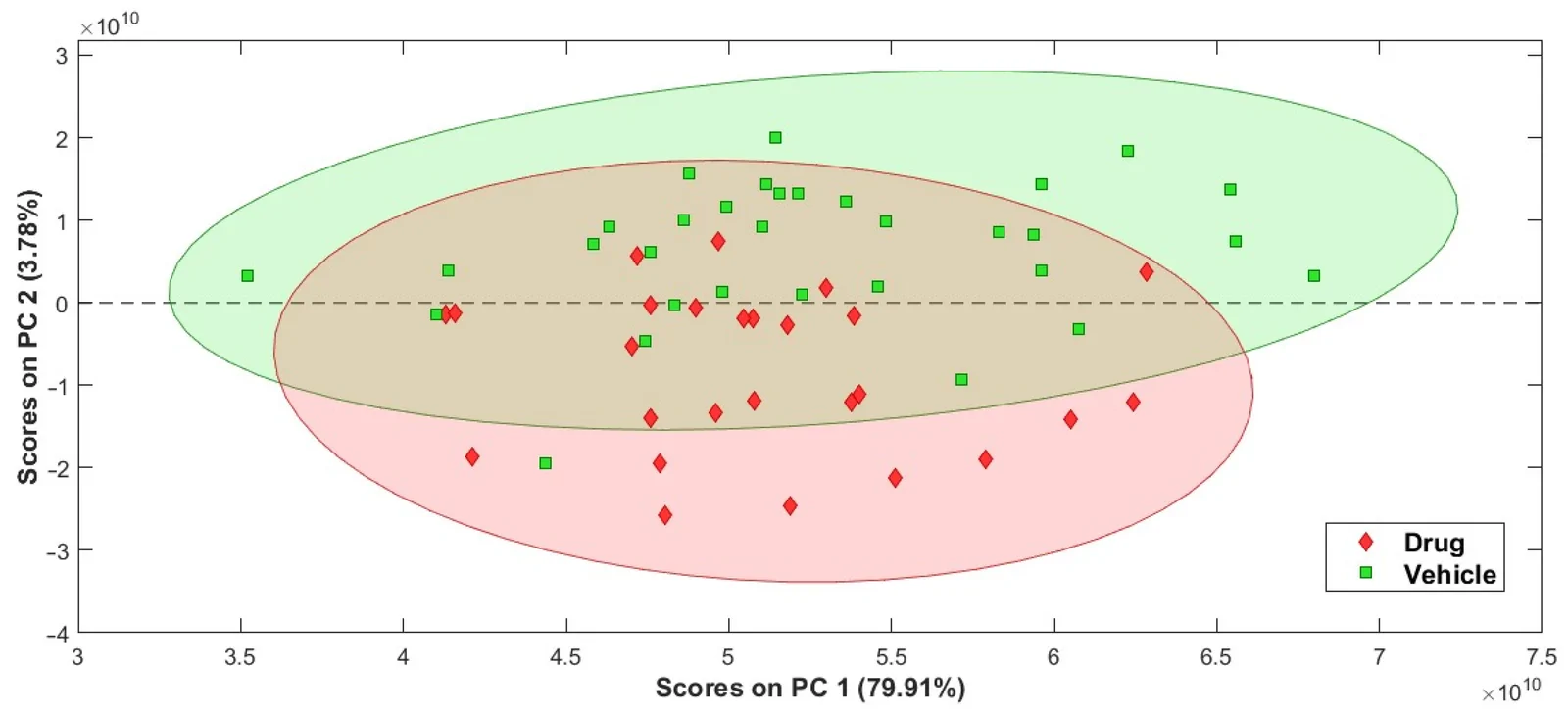
PCA scores plot (PC1 vs. PC2) of vehicle-treated and acutely drug-treated samples. The shaded ellipses summarize the distribution of the samples within each treatment group in the reduced-dimensionality space.

This approach allowed for the emergence of metabolic variations that would have remained masked by the inherent biological variability of urine samples. To evaluate the global metabolic impact of acute treatment, an initial exploratory analysis was conducted on an integrated dataset of 31,217 features, exclusively constituted by vehicle-treated and drug-treated samples following a single administration. The PCA score plot (Figure 2) showed two distinct clusters corresponding to the two experimental groups. Potential outliers in the dataset were identified by inspecting Hotelling’s T^2^ (T^2^) values and Q-residuals, and the relationship between these parameters was visualized using the influence plot.

Subsequently, a PLS-DA model was constructed to investigate the discrimination between vehicle-treated and acutely drug-treated samples. To obtain an approximately unbiased estimate of predictive performance while simultaneously optimizing model complexity, model validation was performed using repeated double cross-validation (rDCV). This procedure repeatedly evaluates the model on independent external test sets while accounting for the variability associated with different data partitions, thereby providing both reliable estimates of predictive performance and a measure of prediction uncertainty [43]. This validation strategy was considered essential because the number of features greatly exceeds the number of samples, a condition that increases the risk of overfitting for the PLS-DA model. In the inner loop, model complexity (number of latent variables) was selected using only the calibration samples, whereas in the outer loop the selected model was applied to external test samples never used for either model building or model selection. The entire procedure was repeated over multiple random partitions, so that the reported figures of merit correspond to out-of-sample predictions and their dispersion reflects model stability. Because multiple samples originated from the same biological subjects, cross-validation partitioning was performed at the animal level. All observations belonging to a given mouse were kept together within either the calibration or validation set in each data split, thereby preventing information leakage between model development and external validation. Within each outer calibration set, model complexity was optimized through the corresponding inner cross-validation loop. The optimal model required 3 ± 1 latent variables and achieved an average accuracy of 85.8 ± 2.0%, with a mean correct classification rate of 85.1 ± 2.2%. Sensitivity and specificity were 78.6 ± 4.0% and 91.7 ± 1.7%, respectively, indicating good overall discriminative performance, although the model showed a greater ability to correctly identify vehicle-treated than drug-treated samples. Nevertheless, given the limited number of animals, this strategy reduces but cannot fully eliminate the risk of overfitting, and the predictive performance reported here should be regarded as an internal estimate requiring confirmation in an independent cohort.

**Figure 3:**
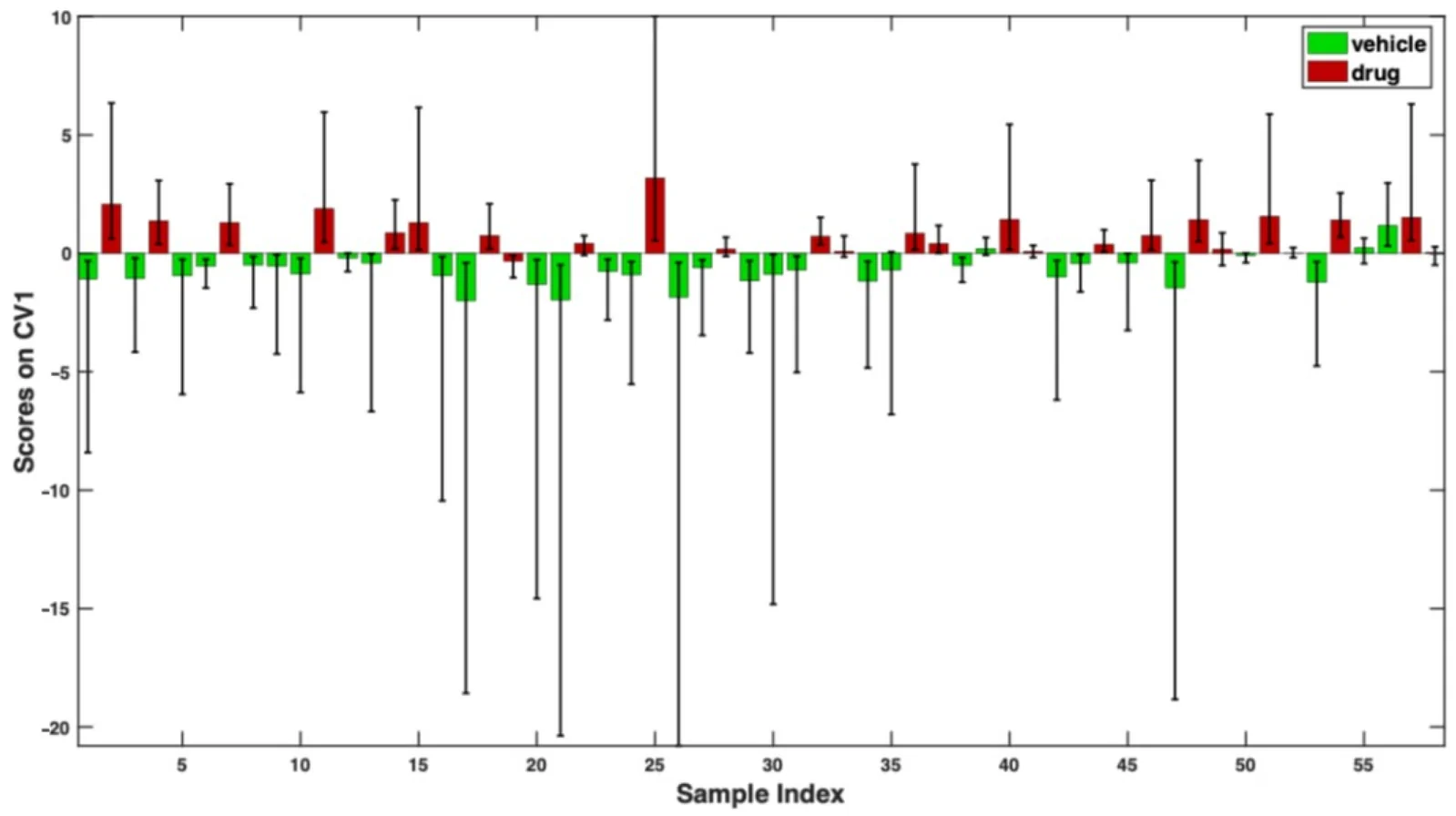
Score plot of Partial Least Squares Discriminant Analysis (PLS-DA) performed on vehicle-treated samples and first drug-treated samples (acute administration).

The corresponding score plot (Figure 3) summarizes the predictions obtained during the outer validation loop of the rDCV procedure. Specifically, each sample was projected only by models for which it belonged to the external validation set and was therefore never used during model calibration or model selection. Consequently, the reported scores represent independent predictions rather than fitted values. Since each sample appears in the outer test set multiple times over the repeated rDCV iterations, a distribution of predicted scores is obtained for every observation. In Figure 3, each point corresponds to the median of this distribution, whereas the error bars denote the corresponding 95% confidence interval, thus providing a graphical representation of prediction uncertainty arising from different calibration subsets. The score plot shows a clear separation between vehicle-treated and acutely drug-treated samples along the first canonical variate (CV1), which represents the latent direction maximizing class discrimination. Drug-treated samples predominantly exhibit positive CV1 scores, whereas vehicle-treated samples are mainly characterized by negative scores. Nevertheless, several samples lie close to the decision boundary, indicating borderline cases associated with greater classification uncertainty. This observation is consistent with the model performance, where sensitivity was slightly lower than specificity, suggesting that a limited number of drug-treated samples exhibited metabolic profiles more closely resembling those of the vehicle group.

To identify the metabolites contributing most strongly to class discrimination, VIP scores were examined [44]. A threshold of VIP > 1.1 was adopted. Since the mean of the squared VIP scores equals 1, a VIP > 1 is conventionally used to identify variables contributing more than average to the model; here, a slightly more restrictive cut-off was applied to retain only the metabolites with the strongest contribution to class separation.

Under this criterion, 1788 discriminant features were identified and subsequently subjected to an annotation workflow following the Metabolomics Standards Initiative (MSI) guidelines. MSI level 1 identifications were assigned when the retention time, fragmentation spectrum, and accurate mass all matched those of an in-house library built from authentic analytical standards, which was the case for 8 metabolites. MSI level 2 annotations were assigned when the analytical standard was not available; in these cases, the accurate mass and fragmentation spectrum were matched against public and commercial databases (GNPS, HMDB, mzCloud, and ChemSpider), yielding 61 putatively annotated metabolites (Table 1). The remaining significant features could not be unambiguously assigned because multiple candidate metabolites were compatible with the observed mass spectral information, no reliable spectral library matches were available, or the acquired MS/MS spectra were insufficient to support a higher level of identification confidence. Regarding the *p*-values reported in Table 1, these were calculated by a two-tailed Student’s *t*-test comparing brorphine-treated and vehicle samples, on the 1788 features selected by PLS-DA (VIP > 1.1). These *p*-values were used, together with the log2 fold changes, to construct the volcano plot, and were corrected for multiple comparisons using the Benjamini–Hochberg procedure for false discovery rate (FDR) control.

As reported in Table 1, the metabolites showing the most significant variations following acute brorphine administration primarily belong to the class of amino acids and their derivatives in agreement with previous studies [32]. In our study, the main alterations were observed in the pathways of tyrosine (tyramine), phenylalanine (2-hydroxyphenylacetic acid) and tryptophan (involving 5-hydroxyindoleacetic acid (5-HIAA), kynurenic acid, 3-hydroxykynurenine, xanthurenic acid, and n-acetyltryptophan) [45]. In addition, alterations were also observed in arginine and proline metabolism, as reflected by urinary biomarkers of muscle and energy turnover, such as creatine and creatinine (level of confidence 1). Beyond the neurochemical profile, the treatment markedly influenced energy and lipid metabolism.

### 3.2. ASCA

Following the exploratory PCA and PLS-DA analyses, the treatment-induced metabolic changes were further investigated using ASCA. In contrast to PCA, which provides an unsupervised overview of the dominant sources of variation, ASCA explicitly exploits the factorial structure of the experimental design to partition the variability associated with each experimental factor and their interactions, thereby facilitating the interpretation of complex multivariate datasets [42]. The ASCA model was applied to the Δ matrix described in the Materials and Methods section, in which each metabolomic profile was expressed relative to the corresponding vehicle sample. Consequently, the analysis focuses specifically on treatment-induced metabolic responses while minimizing baseline inter-individual variability. The decomposition of the Δ matrix according to Equation (1) yielded eight orthogonal components corresponding to the main experimental factors (sex, treatment stage, and sampling interval), their interactions and the residual unexplained variation (Table 2).

**Table 2:** Partitioning of variance in the ASCA model: Explained variance (%) or each model component and corresponding *p*-value evaluated by means of permutation testing.

Effect	Explained Variance (%)	*p*-Value
Sex	3.17	0.00040
Treatment	6.25	0.00010
Sampling time	2.78	0.0014
Sex × Treatment	2.83	0.19
Sex × Sampling time	2.49	0.0046
Treatment × Sampling time	2.72	0.26
Sex × Treatment × Sampling time	2.02	0.88
Residual	77.74	-

Within this framework, the sex effect describes systematic differences in the metabolic response between male and female animals, whereas the treatment stage effect captures the metabolic perturbations associated with the different phases of drug exposure (acute administration, repeated administration and washout). The sampling interval effect reflects the temporal evolution of these metabolic alterations between the 0–12 h and 12–24 h urine collection periods. The interaction terms identify whether the effect of one experimental factor depends on the levels of the others, allowing the identification of sex-dependent responses, treatment-dependent temporal trajectories, and higher-order interactions that characterize the complexity of the metabolic response to brorphine. The residual matrix accounts for the biological and analytical variability not explained by the experimental design.

**Figure 4:**
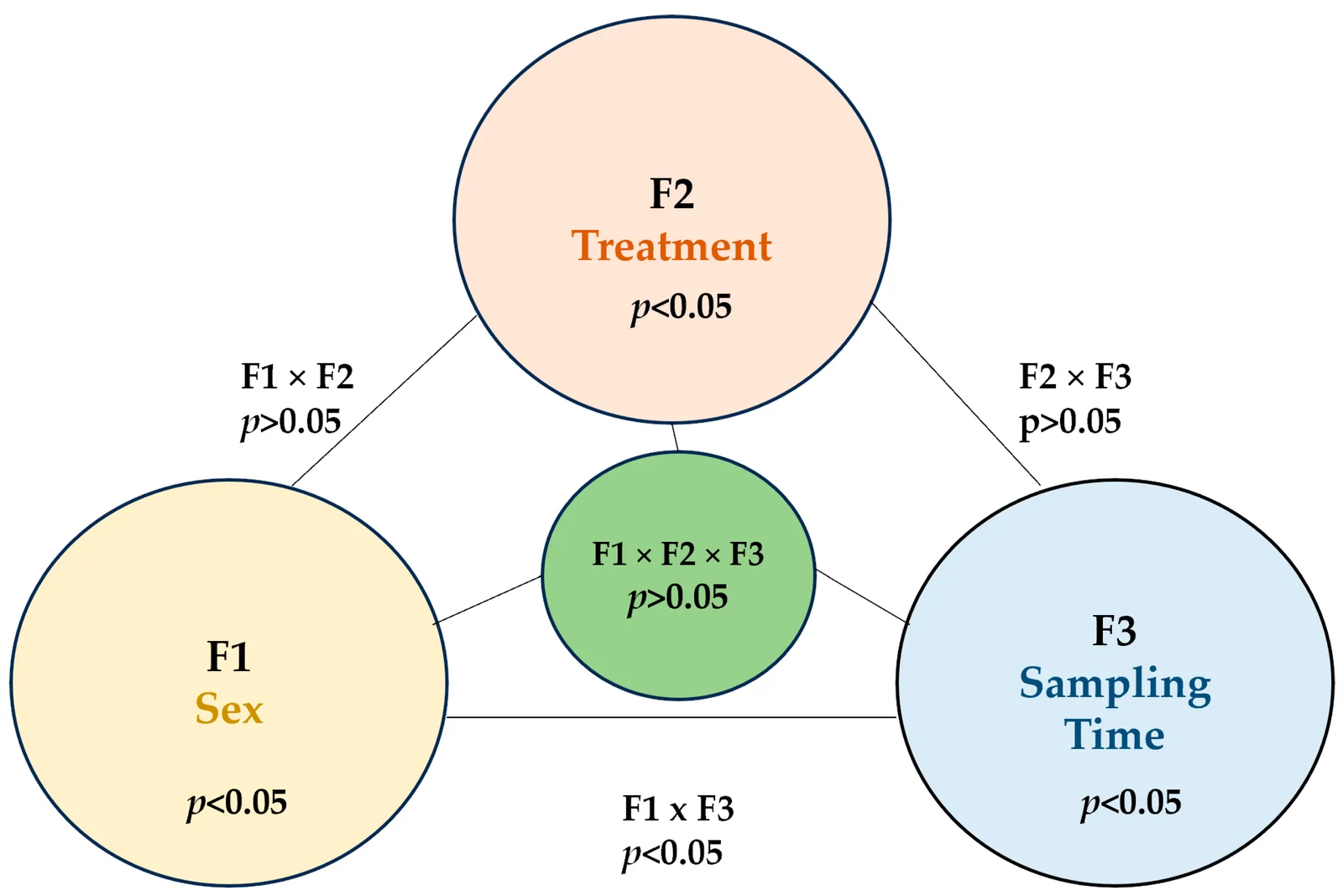
ASCA model diagram illustrating main effects and factor interactions. Main effects (F1: Sex, F2: Treatment, and F3: Collection Time) are represented by circles and show statistical significance (*p* < 0.05), while interaction significance (*p*-values) is reported along the connecting lines.

All three main effects—sex, treatment stage, and sampling interval—were statistically significant (*p* < 0.05), indicating that each factor independently contributed to the observed metabolomic variability (Figure 4). Among the interaction terms, only the interaction between sex and sampling interval reached statistical significance (*p* < 0.05), revealing that the temporal evolution of the metabolic response differed between male and female animals. In contrast, neither the sex × treatment stage nor the treatment stage × sampling interval interactions were significant (*p* > 0.05). These findings indicate that the metabolic differences associated with the various treatment stages (acute administration, repeated administration, and washout) were broadly consistent across sexes and sampling intervals, with no statistical evidence that the effect of treatment stage depended on either factor.

### 3.3. SCA Models and Pathway Analysis

Before discussing the individual experimental effects, it is useful to recall that the SCA decomposition of each effect matrix (Equation (4)) provides complementary information through the corresponding scores and loadings. The score vectors summarize the relationships among the levels of the experimental factor under investigation, whereas the loading vectors identify the metabolites contributing most strongly to the observed differences. Consequently, the interpretation of each significant effect relies on the combined inspection of the associated score and loading plots.

**Figure 5:**
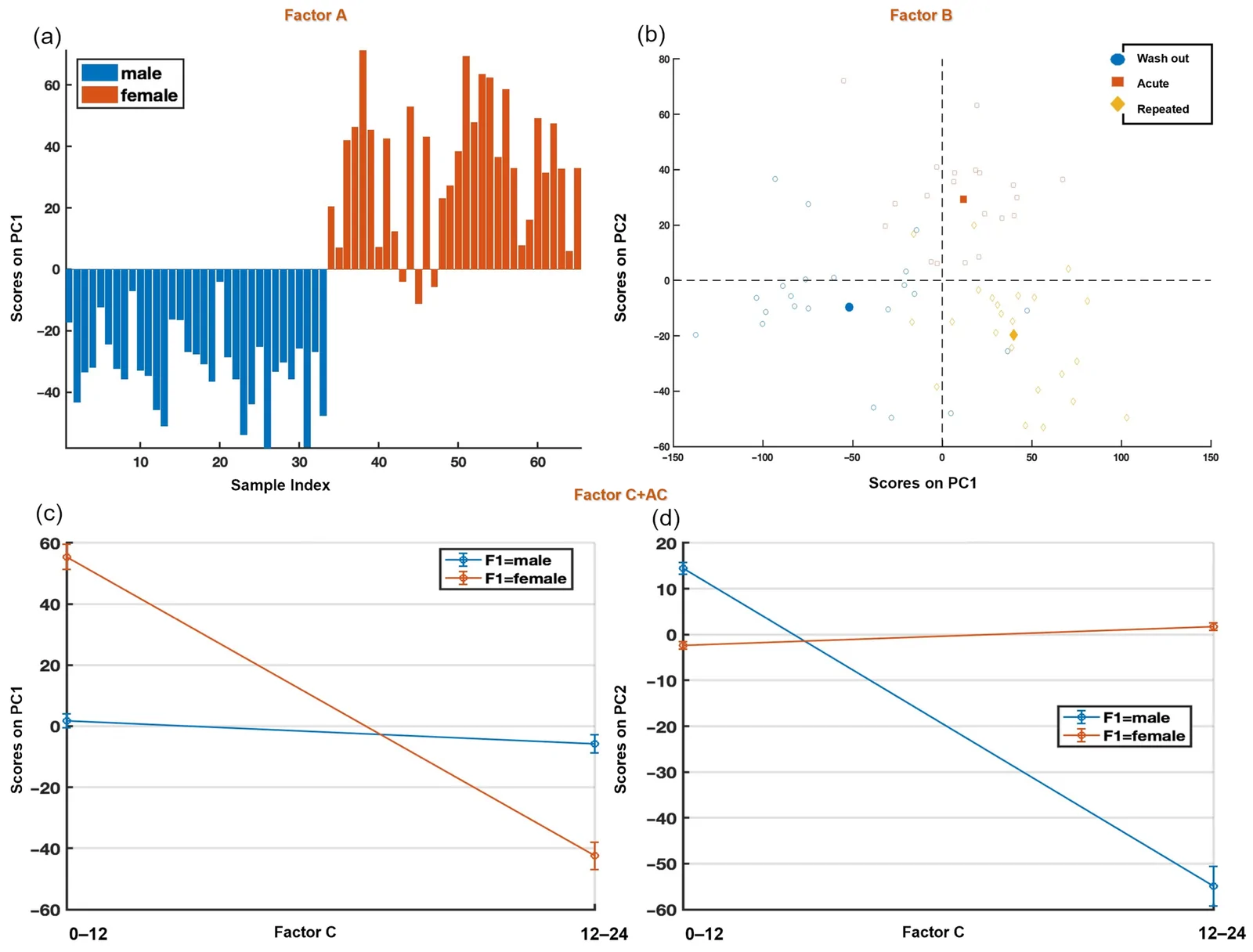
SCA score plots representing the statistically significant experimental factors and their interactions. (**a**) Factor Sex: projection of male and female classes along PC1. (**b**) Factor Treatment: projection of samples along PC1 and PC2 for acute, repeated, and washout administration classes. (**c**,**d**) Sex × Sampling Time interaction: PC1 (**c**) and PC2 (**d**) projections of the sampling time intervals (0–12 h and 12–24 h) partitioned by sex.

Figure 5 summarizes the SCA models obtained for the statistically significant effects, namely sex (factor A), treatment stage (factor B), sampling interval (factor C), and the sex × sampling interval interaction (factor AC). For each effect, the corresponding score plots describe the relationships among the factor levels after removing the contributions of the remaining experimental factors. The sex model (Figure 5a) shows a complete separation between male and female animals along PC1, with males and females occupying opposite regions of the score space. This result indicates that sex represents a major source of variation in the treatment-induced metabolomic response, highlighting marked sex-dependent differences in the metabolic alterations associated with brorphine exposure.

The treatment stage model (Figure 5b) reveals a clear separation between post-treatment samples and those collected during the washout phase along PC1, whereas PC2 further differentiates acute from repeated administration. This pattern suggests that prolonged exposure induces metabolic changes that are distinct from those observed after a single administration, consistent with the occurrence of treatment-dependent metabolic adaptation.

The sampling interval effect (Figure 5c) and, particularly, the significant sex × sampling interval interaction (Figure 5d) provide further evidence that the temporal evolution of the metabolic response differs between male and female animals. Along PC1, females exhibit a larger temporal shift than males, whereas PC2 highlights an additional source of variation that is more pronounced in males. These findings indicate that the temporal trajectories of the treatment-induced metabolomic response differ between the two sexes, suggesting sex-dependent differences in the dynamics of the underlying biochemical processes. Although the score plots alone do not establish the biological mechanisms responsible for these differences, they are compatible with sex-dependent metabolic responses to brorphine, potentially reflecting differences in pharmacokinetic and/or pharmacodynamic behaviour.

**Figure 6:**
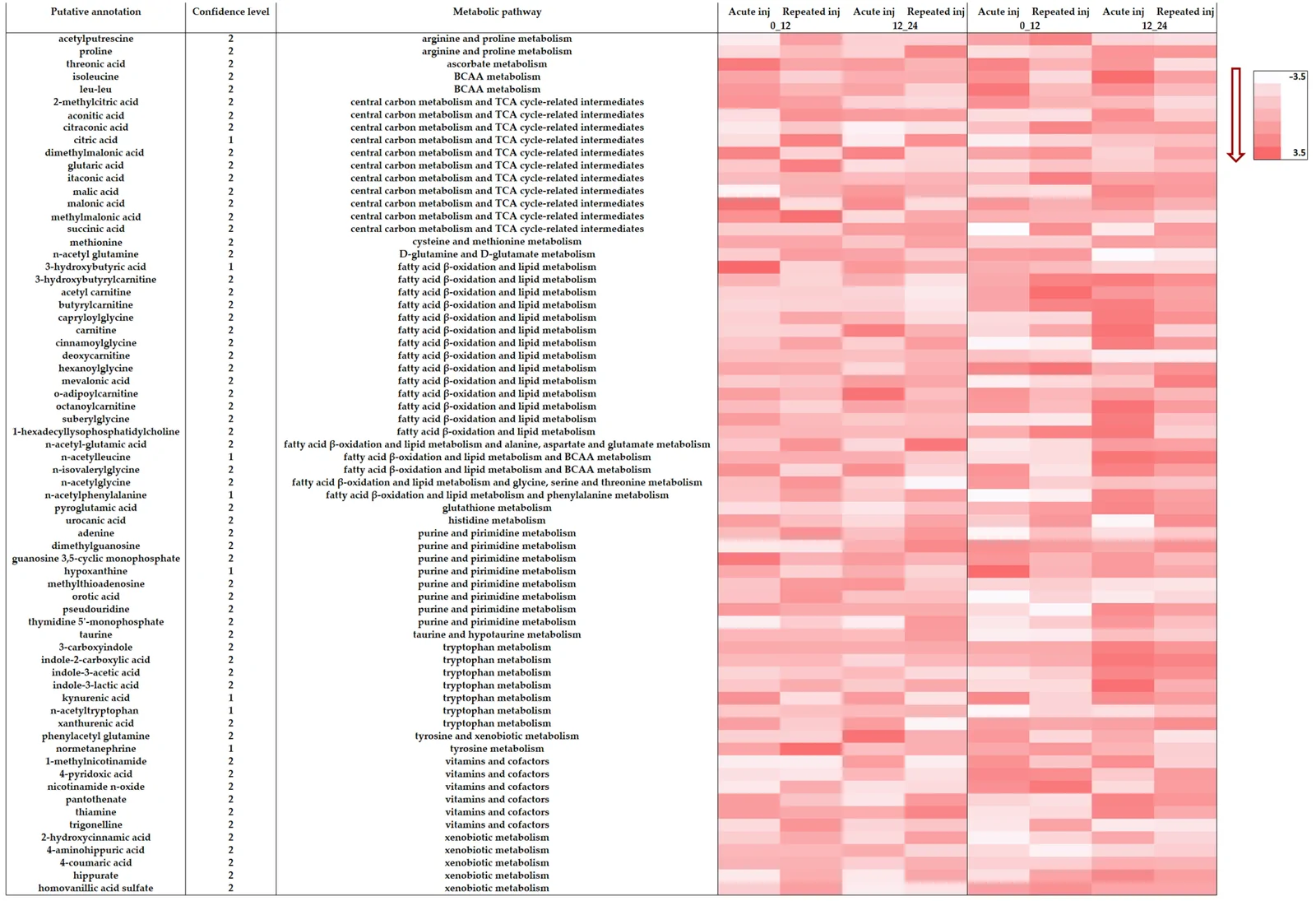
Heatmap of relative variations in urinary metabolites. The metabolites are grouped according to their primary biochemical pathway. Data are stratified by sex (female vs. male) and post-injection time intervals (0–12 h and 12–24 h) following acute (acute inj) and repeated administration (repeated inj). The color scale indicates up-regulation (red) and down-regulation (white) of metabolic levels relative to baseline.

The metabolites contributing most strongly to each significant effect were subsequently identified from the corresponding loading vectors using the same annotation workflow described for the PLS-DA analysis. The resulting metabolite annotations were summarized in the heatmap shown in Figure 6, which provides an overview of the principal metabolic alterations associated with each experimental factor and interaction.

## 4. Discussion

The obtained results showed that brorphine administration markedly affected the metabolome. The use of PLS-DA allowed for a preliminary delineation of the main involved pathways, as evidenced by Table 1.

Consistent with opioid pharmacodynamics, the depletion of GABA, the primary inhibitory neurotransmitter of the central nervous system, integrated with the decrease in homovanillic acid (HVA), the major dopamine metabolite, may reflect a perturbation of dopaminergic and GABAergic neurotransmission induced by substance exposure, and possibly an increased demand on one-carbon metabolism. The reduction in urinary HVA was also observed following repeated brorphine exposure, consistent with the altered dopamine turnover reported after repeated injection with morphine and fentanyl administration.

The depletion of numerous intermediate metabolites in the TCA cycle is consistent with an opioid-induced alteration of mitochondrial energy metabolism [20,45,46], a biochemical signature previously associated with *β*-oxidative stress and common to opioid as well as non-opioid psychoactive substances, including stimulants and alcohol [23]. In fact, according to Li et al. [47], the impairment of acetyl-CoA production affects multiple pathways, such as fatty acid metabolism, one-carbon metabolism and the TCA cycle, which may explain the accumulation of various acyl-intermediates identified in our samples as acylglycines, as well as acetylated metabolites such as acetyl-3-methylhistidine. The concomitant decrease in hippurate and increase in acylglycines levels suggests that glycine may be increasingly recruited for a cellular detoxification mechanism aimed at eliminating acyl-CoA groups that cannot be correctly processed [48]. Furthermore, the altered profile of vitamins and cofactors aligns with this framework, suggesting a possible interference of brorphine with ATP production and cellular respiration [49]. Although most annotations remain putative without authentic reference standards, several of these alterations are consistent with those previously observed in the same animal model following morphine and fentanyl administration [32], which supports their biological plausibility.

However, the PLS-DA approach provided a global overview of the alterations without distinguishing the specific contributions of different experimental factors to the total variance. ASCA was therefore applied to isolate and individually evaluate the effect of sex, treatment, and sampling time, as well as their interactions, on the metabolome response. From a biochemical perspective, ASCA confirmed the findings identified via PLS-DA and, in addition, revealed clear sex-specific divergences and adaptive mechanisms that would otherwise have remained confounded. Consistent with the findings documented by Gabel et al. [50], opioid metabolism is significantly influenced by biological sex: in females, a more rapid biotransformation of the substance correlates with reduced analgesic sensitivity. This evidence is further supported at the molecular level by the immediate metabolic perturbation detected along PC1 (Figure 5c), suggesting a more rapid and intense systemic response in females compared to males, possibly related to a higher efficiency of enzymatic detoxification systems. This divergence indicates that sex-dependent biological factors influence not only the magnitude of the response but also the efficiency of adaptive mechanisms activated during opioid-induced stress.

A significant aspect emerging from the data is the convergence of several altered pathways towards mitochondrial dysfunction for both sexes. Alterations in TCA cycle intermediates and related metabolites, acylcarnitine turnover, ketogenesis, amino acid catabolism, and purine degradation collectively suggest altered mitochondrial bioenergetics; however, the sex (A) and sex-sampling time interaction (AC) factors unveiled contrasting data (Figure 6). The accumulation of acylcarnitines, particularly in males, may reflect enhanced lipid mobilization and points towards incomplete *β*-oxidation and mitochondrial overload; the simultaneous depletion of antioxidant-associated metabolites such as taurine suggests that repeated brorphine administration progressively compromises the cellular capacity to maintain redox balance. ASCA factors A, B and AC, highlighted further differences in detoxification pathways; the involvement of glycine conjugation process [51] was supported by the massive late accumulation (12–24 h) of acylated glycines and hippuric acid in males after the first injection. While this mechanism was rapidly stabilized in females, the persistence of alterations in males even after repeated administration suggests the saturation or inefficiency of the system in the long term.

In females, the increase in 3-hydroxybutyric acid (BHB) represented an effective adaptation, through ketogenesis activation, with a return to homeostasis within 24 h. In contrast, the drastic deficit of BHB in males after the repeated dose signaled energy exhaustion and a possible downregulation of *β*-oxidation [52]. This discrepancy compared to studies on morphine (e.g., Zaitsu et al. [53], Zheng et al. [45]) suggests that the higher potency of brorphine may compromise ketone body production.

Another interesting observation is the reduction in microbial-derived metabolites (hippurate, 4-aminohippuric acid, *p*-cresol glucuronide), which points to the onset of gut dysbiosis following repeated administration [54]. The tardive accumulation of these byproducts, which is greater in males, competes with acyl disposal via the glycine pathway, fueling mitochondrial oxidative stress.

Additionally, ASCA separated sex-dependent responses within purine and pyrimidine metabolism (Figure 6). Although both sexes showed an increase in cyclic guanosine 3′,5′-monophosphate (cGMP), a crucial second messenger in pain modulation and vasodilation that can promote tolerance via the nitric oxide (NO)-cGMP pathway [55], the metabolic response and bioenergetic capacity to sustain such adaptation diverge [50]. The accumulation of hypoxanthine in males suggests accelerated nucleotide degradation. In females, conversely, hypoxanthine decreased significantly after the second injection, suggesting a superior capacity to preserve the purine pool or regulate its degradation. Concurrently, orotic acid, a key precursor for de novo pyrimidine synthesis, shows an increase in females after the second injection, followed by a rapid return to homeostasis, whereas males exhibit a persistent decrease even after repeated injections with no signs of recovery.

The metabolic instability in males is further reflected in the increase in BCAAs (L-isoleucine, Leu-Leu) and pyroglutamic acid and disruptions in tryptophan metabolism. Brorphine treatment induced an evident shunting of tryptophan toward the kynurenine pathway, evidenced by increased levels of xanthurenic acid and kynurenate [47]. This metabolic shift occurred at the expense of serotonine synthesis, as highlighted by the alteration of indole derivatives such as indole-3-acetate and can promote pro-inflammatory processes and oxidative stress. This dynamic is likely fueled by the persistence of brorphine in the CNS of males due to their reduced biotransformation efficiency [50].

A limitation of the present study is the relatively small number of animals (*n* = 5 per sex). Although the sample size was determined a priori based on a moderate effect size derived from our previous work, this number provides limited statistical sensitivity for detecting small effects, particularly in the context of high-dimensional untargeted metabolomics. Accordingly, the metabolomic findings require confirmation in larger, independently powered cohorts. Furthermore, because the same animals were followed longitudinally across experimental phases, the repeated urine samples represent repeated measurements within biological subjects and should not be considered independent biological replicates. These considerations should be taken into account when interpreting the observed sex-associated differences in brorphine pharmacokinetics.

## 5. Conclusions

This study is part of a broader project which points at investigating changes in endogenous urinary metabolite levels as a consequence of the systemic response following opioid intake in order to provide a comprehensive picture of the biochemical response and ultimately develop indirect screening strategies for NPS monitoring. In a previous study we investigated the metabolomics alterations following morphine and fentanyl administration [32] while in this study we provided a comprehensive characterization of sex-specific metabolic alterations induced by acute and repeated brorphine exposure. The application of the ASCA statistical model allowed for elucidating the complexity of HRMS data by isolating systematic differences among experimental factors and identifying the biologically relevant variables responsible for the observed metabolomic variations. Our findings suggest a metabolic divergence linked to sexual diergism consistent with prior observations for morphine and fentanyl. While females exhibit homeostatic resilience supported by high metabolic biotransformation efficiency, males show metabolic features compatible with a systemic bioenergetic malfunction, likely due to greater substance persistence. These findings provide new mechanistic insight into the sex-dependent toxicological effects of brorphine and highlight mitochondrial resilience as a potential determinant of vulnerability to synthetic opioid-induced damage.

The obtained data showed that brorphine induces a transition from acute opioid-mediated metabolic suppression to a compensatory hypermetabolic state characteristic of opioid tolerance. Females successfully engage adaptive mechanisms involving ketogenesis, nucleotide homeostasis and mitochondrial recovery, whereas males exhibit progressive failure of these compensatory responses, resulting in purine depletion, TCA cycle disruption, oxidative stress, gut microbiota dysregulation and neurochemical instability.

Notwithstanding the insights gained, future studies should extend the observation window to evaluate tolerance mechanisms under chronic administration, thereby mapping long-term effects in both sexes. Similarly, investigating the metabolic dynamics associated with withdrawal remains a compelling direction for further research. On an analytical level, we acknowledge the need for complete identification of the significant features and the confirmation of the proposed metabolites using authentic analytical standards.

Nevertheless, the present work provides a highly informative characterization of the effects induced by acute and repeated brorphine exposure, serving as an essential contribution to understanding the toxicological mechanisms necessary to address the current global opioid crisis.

## Figures and Tables

**Table 1:** List of metabolites found to be significant in the PLS-DA analysis and (putatively) identified by matching accurate mass, isotope patterns and/or MS/MS data against spectral and compound databases. The metabolites are grouped according to their primary biochemical pathway: alanine, aspartate and glutamate metabolism (a), arginine and proline metabolism (b), glycine, serine and threonine metabolism (c), branched-chain amino acids (BCAA) metabolism (d), central carbon metabolism and TCA cycle-related intermediates (e), fatty acid *β*-oxidation and lipid metabolism (f), histidine metabolism (g), lysine degradation (h), phenylalanine metabolism (i), purine and pyrimidine metabolism (j), taurine and hypotaurine metabolism (k), tryptophan metabolism (l), tyrosine metabolism (m), vitamins and cofactors (n), xenobiotic metabolism (o).

Putative Annotation	Metabolic Pathway	Formula	Retention Time (min)	Analysis	log2FC	*p*-Value	Mass Error	Level of Confidence
4-acetamidobutanoate	a	C_6_H_11_NO_3_	2.29	RP−	0.60	6.48 × 10^−3^	−4.06	2
creatinine	b	C_4_H_7_N_3_O	3.08	HC+	0.24	1.72 × 10^−2^	−0.04	1
GABA	b	C_4_H_9_NO_2_	5.99	HC+	−1.03	6.94 × 10^−4^	0.76	2
proline	b	C_5_H_9_NO_2_	1.53	RP+	−1.37	5.40 × 10^−4^	0.68	2
urea	b	CH_4_N_2_O	1.09	HC+	−0.24	5.76 × 10^−4^	3.37	2
creatine	b/c	C_4_H_9_N_3_O_2_	6.72	HC+	0.99	7.68 × 10^−4^	0.03	1
l-isoleucine	d	C_6_H_13_NO_2_	1.53	RP+	1.28	9.23 × 10^−4^	−0.85	2
prolylleucine	d	C_11_H_20_N_2_O_3_	2.15	RP+	1.00	1.94 × 10^−3^	−1.69	2
leu-ile	d	C_12_H_24_N_2_O_3_	6.29	RP+	2.99	1.42 × 10^−6^	−1.83	2
citric acid	e	C_6_H_8_O_7_	1.34	RP−	−1.05	5.70 × 10^−4^	−3.30	1
2-oxoglutaric acid	e	C_5_H_6_O_5_	1.12	RP−	−0.75	9.92 × 10^−3^	−3.61	2
dl-malic acid	e	C_4_H_6_O_5_	1.00	RP−	−1.06	4.56 × 10^−3^	−2.90	2
glutaric acid	e	C_5_H_8_O_4_	2.56	RP−	−0.81	1.63 × 10^−2^	−3.28	2
dimethylmalonic acid	e	C_5_H_8_O_4_	3.07	RP−	0.82	6.60 × 10^−4^	−3.16	2
itaconic acid	e	C_5_H_6_O_4_	1.92	RP−	0.76	8.10 × 10^−3^	−2.54	2
succinate	e	C_4_H_6_O_4_	0.718/1.497	HC−/RP+	−0.68	3.75 × 10^−2^	−2.23	2
3-hydroxybutanoic acid	f	C_4_H_8_O_3_	0.87	HC−	3.09	4.92 × 10^−3^	−0.67	1
3-hydroxy-3-methylglutaric acid	f	C_6_H_10_O_5_	1.83	RP−	1.46	2.01 × 10^−5^	−4.25	2
carnitine	f	C_7_H_15_NO_3_	0.85	RP+	−0.82	5.54 × 10^−2^	−1.12	2
crotonic acid	f	C_4_H_6_O_2_	2.65	RP−	2.27	2.62 × 10^−2^	−3.46	2
mevalonic acid lactone	f	C_6_H_10_O_3_	2.37	RP+	2.11	1.76 × 10^−3^	−0.66	2
malonic acid	f	C_3_H_4_O_4_	1.15	HC−	2.28	6.91 × 10^−5^	−0.55	2
n-butyrylglycine	f	C_6_H_11_NO_3_	2.77	RP−	1.04	1.11 × 10^−5^	−3.86	2
n-tigloylglycine	f	C_7_H_11_NO_3_	4.90	RP−	1.68	2.65 × 10^−7^	−4.46	2
phenylcarbamoylglycine	f	C_9_H_10_N_2_O_3_	0.96	HC−	−0.55	1.54 × 10^−2^	0.76	2
2-methylbutyrylglycine	f	C_7_H_13_NO_3_	4.73	RP−	0.85	2.10 × 10^−2^	−4.39	2
hexahydrohippurate or cyclohexanoylglycine	f	C_9_H_15_NO_3_	7.19	RP−	1.39	5.53 × 10^−4^	−3.65	2
hexanoylglycine	f	C_8_H_15_NO_3_	6.68	RP−	0.98	2.27 × 10^−3^	−4.05	2
n-isovalerylglycine	f/d	C_7_H_13_NO_3_	4.95	RP+	1.03	4.11 × 10^−3^	−0.82	2
propionylcholine	e	C_8_H_19_NO_2_	6.91	HC+	0.80	3.67 × 10^−2^	0.12	2
glycine	c	C_2_H_5_NO_2_	1.45	RP+	1.51	4.71 × 10^−3^	−0.95	2
1-methylhistamine	g	C_6_H_11_N_3_	0.76	RP+	0.91	1.46 × 10^−3^	−0.84	2
acetyl-3-methylhistidine	g	C_9_H_13_N_3_O_3_	1.14	RP+	1.21	1.79 × 10^−4^	−1.24	2
aminoadipate	h	C_6_H_11_NO_4_	1.33	RP−	2.44	9.44 × 10^−5^	−4.07	2
2-hydroxyphenylacetic acid	i	C_8_H_8_O_3_	6.43	RP−	−0.37	2.42 × 10^−2^	−4.37	1
2-deoxyadenosine	j	C_10_H_13_N_5_O_3_	1.70	HC+	0.74	7.56 × 10^−4^	−0.95	1
hypoxanthine	j	C_5_H_4_N_4_O	1.39	HC+	0.68	1.16 × 10^−3^	0.25	1
7-methylguanine	j	C_6_H_7_N_5_O	1.64	RP+	0.74	6.65 × 10^−3^	−0.90	2
9-methyluric acid	j	C_6_H_6_N_4_O_3_	1.63	RP−	1.47	2.86 × 10^−7^	−3.94	2
acadesine	j	C_9_H_14_N_4_O_5_	1.48	HC+	0.87	7.38 × 10^−5^	−1.03	2
allantoin	j	C_4_H_6_N_4_O_3_	0.82	HC−	−0.28	2.15 × 10^−3^	−4.13	2
cytosine	j	C_4_H_5_N_3_O	2.86	HC+	−0.87	6.62 × 10^−5^	0.51	2
methylcytidine	j	C_10_H_15_N_3_O_5_	2.14	HC+	0.58	2.57 × 10^−2^	−1.14	2
methylguanidine	j	C_2_H_7_N_3_	4.28	HC+	1.25	3.08 × 10^−5^	0.37	2
methylguanosine	j	C_11_H_15_N_5_O_5_	4.63	RP+	0.68	1.57 × 10^−2^	−1.56	2
orotic acid	j	C_5_H_4_N_2_O_4_	1.53	HC−	−1.23	2.54 × 10^−3^	−3.15	2
xanthine	j	C_5_H_4_N_4_O_2_	1.76	RP−	0.99	1.96 × 10^−2^	−4.16	2
taurine	k	C_2_H_7_NO_3_S	2.86	HC−	−0.43	5.29 × 10^−3^	−2.29	2
kynurenic acid	l	C_10_H_7_NO_3_	2.58	HC+	0.26	3.19 × 10^−2^	0.47	1
3-carboxyindole	l	C_9_H_7_NO_2_	6.82	RP+	1.97	1.31 × 10^−2^	−1.08	2
5-HIAA	l	C_10_H_9_NO_3_	6.66	RP+	−0.50	5.64 × 10^−2^	−0.69	2
indole-3-carboxaldehyde	l	C_9_H_7_NO	7.93	RP+	−1.49	5.53 × 10^−3^	−0.54	2
n-acetyltryptophan	l	C_13_H_14_N_2_O_3_	0.84	HC+	−1.55	1.72 × 10^−4^	−0.85	2
xanthurenic acid	l	C_10_H_7_NO_4_	5.90	RP−	1.10	2.68 × 10^−3^	−2.70	2
3-hydroxykynurenine	l	C_10_H_12_N_2_O_4_	6.07	RP−	−0.58	2.09 × 10^−2^	−2.88	2
tyramine	m	C_8_H_11_NO	2.73	HC+	−0.82	2.51 × 10^−4^	−0.06	2
4-pyridoxate	n	C_8_H_9_NO_4_	3.27	RP−	0.48	8.79 × 10^−3^	−2.44	2
biopterin	n	C_9_H_11_N_5_O_3_	1.93	RP+	1.41	7.22 × 10^−4^	−1.72	2
riboflavin	n	C_17_H_20_N_4_O_6_	1.29	HC+	−0.84	1.05 × 10^−6^	2.16	2
threonic acid	n	C_4_H_8_O_5_	0.87	RP−	0.49	3.07 × 10^−4^	−2.87	2
trigonelline	n	C_7_H_7_NO_2_	6.76	HC+	−0.90	2.37 × 10^−4^	−0.11	2
1-methylnicotinamide	n	C_7_H_9_N_2_O	6.27	HC+	−0.64	9.42 × 10^−3^	0.43	2
3-hydroxycinnamic acid	o	C_9_H_8_O_3_	7.26	RP−	−1.08	1.79 × 10^−2^	−3.28	2
hippurate	o	C_9_H_9_NO_3_	5.93	RP−	−0.56	9.93 × 10^−4^	−3.95	2
homovanillic acid sulfate	o	C_9_H_10_O_7_S	5.38	RP−	−0.72	1.88 × 10^−2^	−1.85	2
*p*-cresol glucuronide	o	C_13_H_16_O_7_	6.61	RP−	0.94	2.85 × 10^−3^	−0.68	2
*p*-cresol sulfate	o	C_7_H_8_O_4_S	6.43	RP−	0.81	1.27 × 10^−2^	−3.50	2
phenylacetylglutamine	o	C_13_H_16_N_2_O_4_	1.71	HC+	0.61	1.49 × 10^−2^	−1.23	2
aminohippuric acid	o	C_9_H_10_N_2_O_3_	0.75	HC−	−1.00	4.59 × 10^−7^	−3.51	2

## Data Availability

The data presented in this study are available on request from the corresponding author.

## Protocol Registration Statement

No formal study protocol, including a predefined research question, key design features, and analysis plan, was registered prior to the start of the study.
